# Response to Letter to the Editor, “Sarcopenic Obesity: An Emerging Public Health Problem, But an Answer Appears to Be Available”

**DOI:** 10.14336/AD.2021.1120-1

**Published:** 2022-06-01

**Authors:** Tong Ji, Yun Li, Lina Ma

**Affiliations:** Department of Geriatrics, Xuanwu Hospital, Capital Medical University, National Research Center for Geriatric Medicine, Beijing 100053, China

**To the editor,**

We appreciate the valuable and thoughtful suggestions and comments made by Dr. Machado regarding our published work [1]. We are pleased to accept the recommendations and put forward our ideas.

While it has great potential, the application and influence of creatine supplementation on sarcopenic obesity (SO) in older adults remain little-known because there are relatively few studies in this field. As Dr. Marco observes [2], creatine supplementation coupled with resistance training improves measures of lean tissue mass, strength, and physical performance in older adults compared with placebo [3-6]. A recent systematic review suggested creatine supplementation in conjunction with resistance training led to more reduction in fat mass and percentage of body fat than placebo and resistance training [7].

Judging from the current literature, creatine supplementation appears to be a potential nutritional strategy for preventing and treating SO. However, there are a lot of challenges in applying creatine supplementation to clinical practice.

First, the effects of creatine supplementation in SO and creatine supplementation alone on fatty tissue biology in older adults remain to be established. Two recent large trials have shown that long-term creatine supplementation alone seems to have little or no effect on lean mass [8, 9].

Second, it is difficult to control creatine supplementation dosing strategy accurately for every aged patient because the current research heterogeneous. Creatine-loading followed by a lower daily dosage of creatine supplementation (≤5 g) could increase upper-body strength vs. placebo, according to a new systematic review [4]. This review showed that a higher daily dosage of creatine supplementation (>5 g) after the loading phase could increase lower-body strength, and creatine supplementation only on resistance training days increased measures of lean tissue mass and strength vs. placebo.

Last but not least, although creatine supplementation is one of the most studied dietary supplements, has fewer side effects than others, and is considered safe, the physiological function of the aged is considered to have declined, especially in those with various comorbidities, polypharmacy, and renal malfunction; therefore, we should be cautious in the use of creatine supplementation in clinical treatment.

Collectively, creatine supplementation accompanied by resistance training appears to be a potential and effective intervention in older adults. Practice is the sole criterion for testing truth; therefore, future research is needed with larger and long-term clinical trials involving older adults with various illnesses and polypharmacy. We thank Dr. Marco very much for the advice.
